# Species Differentiation of Chinese *Mollitrichosiphum* (Aphididae: Greenideinae) Driven by Geographical Isolation and Host Plant Acquirement

**DOI:** 10.3390/ijms130810441

**Published:** 2012-08-21

**Authors:** Ruiling Zhang, Xiaolei Huang, Liyun Jiang, Fumin Lei, Gexia Qiao

**Affiliations:** 1 Key Laboratory of Zoological Systematics and Evolution, Institute of Zoology, Chinese Academy of Sciences, Beijing 100101, China; E-Mails: zhangrl_06@126.com (R.Z.); huangxl@ioz.ac.cn (X.H.); jiangliyun@gmail.com (L.J.); leifm@ioz.ac.cn (F.L.); 2 Graduate University of Chinese Academy of Sciences, Beijing 100049, China

**Keywords:** aphid, divergence time, geographical isolation, host plant, *Mollitrichosiphum*, phylogeny, Qinghai-Tibetan Plateau, southern China, speciation

## Abstract

The impact of both the uplift of the Qinghai-Tibetan Plateau (QTP) and the separation of the Taiwan and Hainan Islands on the evolution of the fauna and flora in adjacent regions has been a topic of considerable interest. *Mollitrichosiphum* is a polyphagous insect group with a wide range of host plants (14 families) and distributions restricted to Southeast Asia. Based on the mitochondrial Cytochrome C Oxidase Subunit I (COI) and Cytochrome b (Cytb) genes, the nuclear elongation factor-1α (EF-1α) gene, and the detailed distribution and host plant data, we investigated the species differentiation modes of the Chinese *Mollitrichosiphum* species. Phylogenetic analyses supported the monophyly of *Mollitrichosiphum*. The divergence time of *Mollitrichosiphum tenuicorpus* (*c*. 11.0 mya (million years ago)), *Mollitrichosiphum nandii* and *Mollitrichosiphum montanum* (*c*. 10.6 mya) was within the time frame of the uplift of the QTP. Additionally, basal species mainly fed on Fagaceae, while species that fed on multiple plants diverged considerably later. Ancestral state reconstruction suggests that Fagaceae may be the first acquired host, and the acquisition of new hosts and the expansion of host range may have promoted species differentiation within this genus. Overall, it can be concluded that geographical isolation and the expansion of the host plant range may be the main factors driving species differentiation of *Mollitrichosiphum*.

## 1. Introduction

Southern China is characterized by a complex topography and represents the major biodiversity hotspots [1]. This region includes several mountain chains (*i.e.*, the Wuyi, Nanling and Hengduan Mountains) and two islands (Taiwan and Hainan). The uplift of the Qinghai-Tibetan Plateau (QTP), which strongly influenced the environment of the plateau and its surrounding areas, as well as the climate in Asia and across the globe, was one of the most important events in the Earth’s geological history [2–4]. The recent violent uplifts of the QTP during the Quaternary initiated the formation of mountain and valley glaciers in many areas [2,5,6], both the Taiwan and Hainan Islands were repeatedly connected and separated with the mainland following the glacial and interglacial cycle over the past million years [7]. It has been suggested that these events greatly influenced the biogeography of the fauna and flora in this region and the surrounding areas [7–9].

Studies based on distribution data have indicated that the Hengduan Mountains, north of the Sichuan and Yunnan Province, and the Hainan and Taiwan Islands are diversity centers for birds, amphibians, insects and plants [10–15]. These studies, which are based on distribution records, described the primary spatial diversity patterns in southern China. However, research based on phylogeny and a time-calibrated framework is required to understand the evolutionary history of the species diversity in this region. To date, some phylogeographical studies have been performed in southern China, especially focused on plants [16–18], mammals [19], birds [20–22] and reptiles [23]. However, few of these reports discussed the biogeographical patterns beyond the species level and across a wide geographical range.

The aphid genus *Mollitrichosiphum* (Aphididae, Greenideinae) was described by Suenaga in 1934, based the description on the numerous transverse ridges on the hind tibiae, six elongated antennal segments and a pointed ultimate rostral segment. *Mollitrichosiphum* contains 18 known species, based on the different lengths and distributions of the setae on the antenna and the shape of the radial sector. This genus is divided into two subgenera, namely, *Mollitrichosiphum* and *Metatrichosiphon* [24,25]. The hosts of this genus belong to the plant families Fagaceae and Betulaceae, as well as to 12 other families (Table 1). While the host plant of *Mollitrichosiphum* (*Metatrichosiphon*) *kazirangi* is unknown, it has been demonstrated that 14 species of *Mollitrichosiphum* feed on Fagaceae. Three (out of four) species of the subgenus *Mollitrichosiphum* feed exclusively on Fagaceae, while the species of the subgenus *Metatrichosiphon* display higher host diversity. For example, *Mollitrichosiphum* (*Metatrichosiphon*) *rhusae* and *Mollitrichosiphum* (*Metatrichosiphon) nandii* infest plants belonging to four families (Anacardiaceae, Proteaceae, Meliaceae and Fagaceae; Betulaceae, Lauraceae, Sapindaceae and Fagaceae, respectively), and *Mollitrichosiphum* (*Metatrichosiphon*) *montanum* and *Mollitrichosiphum* (*Metatrichosiphon*) *nigrum* (Betulaceae, Juglandaceae and Fagaceae; Sabiaceae, Simaroubaceae and Elaeagnaceae, respectively) feed on plants belonging to three different families.

*Mollitrichosiphum* is mainly distributed throughout Southeast Asia (Table S1). The subgenus *Mollitrichosiphum* is restricted to Nepal, northeastern India (Sikkim) and the southern edge of the QTP, which is characterized by a notably complex mountainous topography. Most species of the subgenus *Metatrichosiphon* are primarily distributed in the eastern region of Southeast Asia. Twelve known species in China can be assigned to three distribution patterns: widespread throughout southern China (*Mollitrichosiphum* (*Mollitrichosiphum*) *tenuicorpus*), the Himalaya-Hengduan Mountains (*Mollitrichosiphum* (*Metatrichosiphon*) *montanum* and *Mollitrichosiphum* (*Metatrichosiphon*) *nandi*) and southeast of China (*Mollitrichosiphum* (*Metatrichosiphon*) *rhusae*, *Mollitrichosiphum* (*Metatrichosiphon*) *luchuanum*, *Mollitrichosiphum* (*Metatrichosiphon*) *nigrofasciatum*, *Mollitrichosiphum* (*Metatrichosiphon*) *nigrum* and *Mollitrichosiphum* sp.). These patterns provide the opportunity to determine the relative effects of geographical isolation caused by both the uplift of the QTP and the separation of the islands on the differentiation of these species.

Previous studies of this genus were alpha-taxonomic, and species descriptions based on morphology [25–29]. Zhang *et al*. [30] primarily investigated the phylogenetic relationships of seven Chinese *Mollitrichosiphum* species based on the mitochondrial Cytochrome C Oxidase Subunit I (COI) and Cytochrome b (Cytb) genes. Their results indicated that the widespread species, *M. tenuicorpus*, may consist of three cryptic species, which are isolated by the Tibetan Plateau and Lancang River. However, these results require further investigation with additional data to elucidate the relationship between the species of this genus and the effects of host plants on species divergence.

Based on the geographic distribution and host plant use data of *Mollitrichosiphum*, we hypothesized that both geographical isolation and host plant diversity may have played important roles in species differentiation of *Mollitrichosiphum* in southern China. To test this hypothesis, we used extensive sampling and combined mitochondrial (COI, Cytb) and nuclear (elongation factor-1α, EF-1α) data to reconstruct the phylogenetic relationships of the Chinese *Mollitrichosiphum* species. We also estimated the divergence times of *Mollitrichosiphum* using a Bayesian approach to evaluate the potential relationship between species divergence, geological events and host plant evolution.

## 2. Results and Discussion

### 2.1. Results

#### 2.1.1. Phylogenetic Analyses

**Figure 1:**
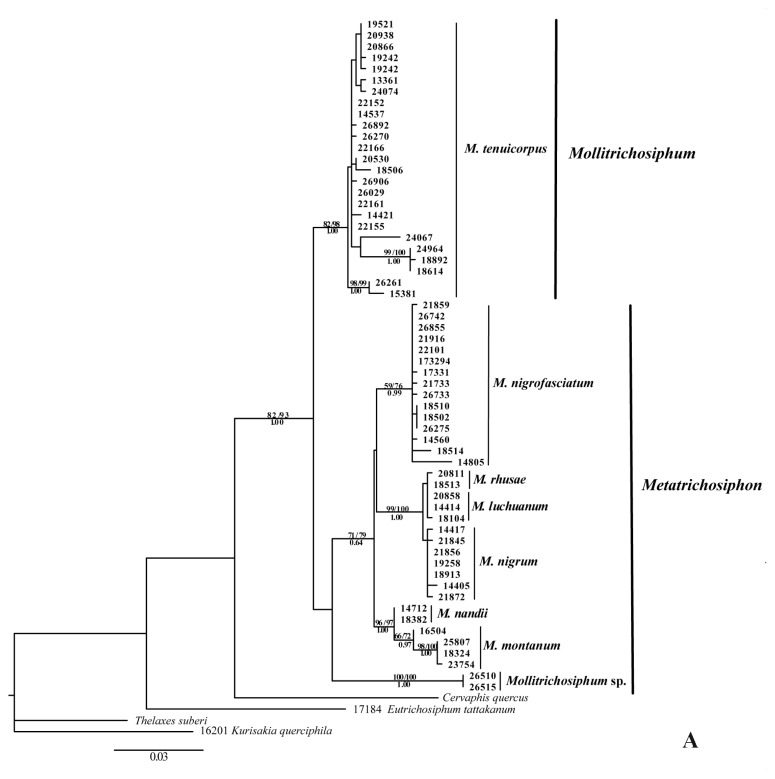

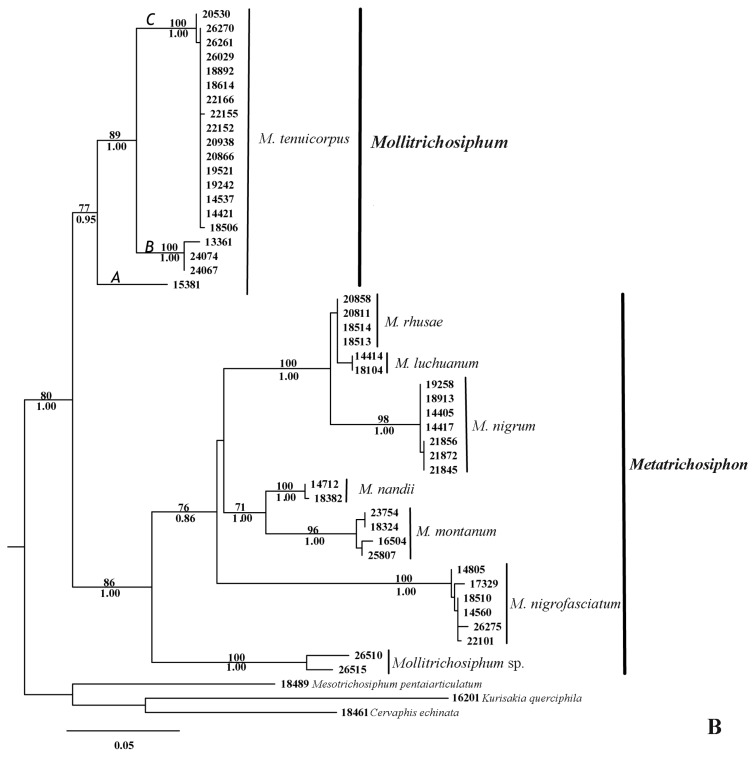
Phylogenetic relationships of *Mollitichosiphum* species based on Maximum likelihood analysis of the EF-1α sequences (**A**) and combined dataset of Cytochrome C Oxidase Subunit I (COI), Cytochrome b (Cytb) and Elongation Factor-1α (EF-1α) sequences (**B**). Bootstrap values (≥50%) for maximum parsimony (MP), maximum likelihood (ML) are shown above the branches, Bayesian posterior probabilities (≥50%) are shown below the branches. In Figure 1B, italic scripts of A, B and C represent the three clades in *M. tenuicorpus*.

We used mitochondrial gene COI, Cytb, and nuclear gene EF-1α to reconstruct the phylogenetic relationships of *Mollitrichosiphum*. All sequences were submitted to GenBank (see Table 2 for accession numbers). The detailed characters of the individual sequences and the combined dataset are listed in Table 3. The monophyly of the sampled *Mollitrichosiphum* species was supported by all phylogenetic analyses. The results based on the combined dataset of COI and Cytb were similar to that of a previous study [30] (data not shown here). Phylogenetic trees based on EF-1α sequences and the combined dataset of the EF-1α and mitochondrial genes are shown in Figure 1. In the phylogenetic analyses based on the EF-1α gene, four representatives of Thelaxinae, Cervaphidini and Greenideini were used to root the trees. Maximum parsimony (MP), maximum likelihood (ML) and Bayesian (BI) analyses yielded congruent trees, and the tree obtained from the ML analysis is shown (Figure 1A). The monophyly of the genus *Mollitichosiphum* was highly supported (MP/ML/BI: 82/93/1.00). Three samples of Greenideini, Cervaphidini and Thelaxinae were used as outgroups in the Bayesian and ML analyses of the combined dataset. The combined dataset also supported the monophyly of this genus (ML/BI: 80/1.00) and of most species (Figure 1B). All analyses supported the sister relationship between *M. nandii* and *M. montanum*. Interestingly, the samples of *M. luchuanum* and *M. rhusae* clustered together and formed a paraphyletic clade in the phylogenetic tree based on the EF-1α, while the two species could be distinguished in the trees based on combined datasets of the two mitochondrial genes as well as the total three genes. Moreover, the position of *M. nigrum* as a sister clade of *M. luchuanum* and *M. rhusae* is well-supported by all analyses. The basal position of *Mollitrichosiphum* sp. in the subgenus *Metatrichosiphon* is well-supported in the combined dataset, although the supports were lower than 50% with the EF-1α gene. The phylogenetic relationships of *M. nigrofasciatum* with other species are uncertain. In the EF-1α tree, *M. nigrofasciatum* grouped together with *M. nigrum*, *M. rhusae* and *M. luchuanum* (bootstrap values and posterior probabilities were all lower than 50%), and based on the combined dataset, this species forms a sister clade with *M. nigrum*, *M. rhusae*, *M. luchuanum*, *M. montanum* and *M. nandii* (ML/BI: 76/0.86). In the phylogenetic analyses based on the combined dataset, the samples of *M. tenuicorpus* were subdivided into three major clades (Figure 1B), which was also supported by the results obtained with EF-1α to some extent (Figure 1A).

#### 2.1.2. Divergence Time and Geographical Distribution

**Figure 2:**
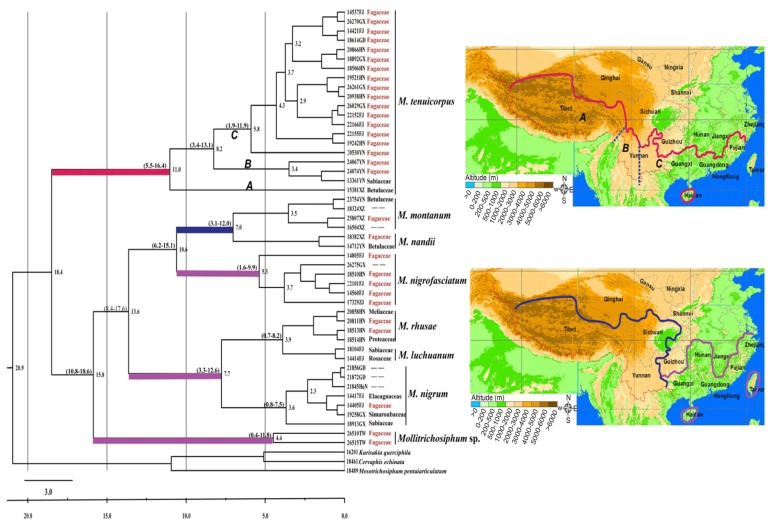
A chronogram of the Chinese *Mollitrichosiphum* species based on the combined COI and Cytb genes. Median age estimates are shown behind nodes (mya). Italic scripts of A, B and C represent three clades in *M. tenuicorpus*. Clades were marked with colours corresponding to the distributions of samples, and colour boundaries indicate the distribution ranges of clades. Host plant information was given following the sample names, with Fagaceae indicated in red, “--” represents host plants that are unknown. Locality abbreviated in capital letters: XZ, Tibet; YN, Yunnan; HN, Hainan; GX, Guangxi; FJ, Fujian; GD, Guangdong; ZJ, Zhejiang; HuN, Hunan; SC, Sichuan.

The divergence times were estimated based on the mitochondrial and nuclear data separately, and the mean age estimates of most nodes were largely consistent. The molecular dating results indicate that clade A within *M. tenuicorpus* diverged at approximately 11.0 mya (95% Highest Posterior Density, HPD: 5.5–16.4 mya) (Figure 2), while clades B and C diverged at 8.2 mya (HPD: 3.7–13.1 mya). The ancestors of *Mollitrichosiphum* sp. and the other species of subgenus *Metatrichosiphon* diverged at approximately 15.8 mya (HPD: 10.8–18.6 mya). The most recent common ancestors (MRCAs) of the other six species were 13.6 mya (HPD: 8.4–17.6 mya). Divergence among the individuals of six species (*M. rhusae*, *M. nigrum*, *M. nandii*, *M. luchuanum*, *M. nigrofasciatum* and *M. montanum*) were between 10.6 mya (HPD: 6.2–15.1 mya) and 3.9 mya (HPD: 0.7–8.2 mya), with *M. nigrofasciatum* exhibiting the earliest within species divergence (5.3 mya), and MRCAs of *M. montanum* and *M. nandii* was estimated at 7.0 mya (HPD: 3.1–12.0 mya). *M. tenuicorpus* widespread from northeastern India, southern China, Thailand, Indonesia, Japan and Korea (Table S1); ranges of other seven species appear to be constrained. *M. montanum* and *M. nandii* are distributed on the south edge of the QTP and Hengduan Moutians; *M. luchuanum*, *M. nigrum*, *M. nigrofasciatum*, *M. rhusae* and *Mollitrichosiphum* sp. are limited to southeastern China. Among them, *M. rhusae* and *Mollitrichosiphum* sp. are distributed in the Hainan and Taiwan islands, respectively. Distributions of these species were mapped roughly in Figure 2.

#### 2.1.3. Ancestral State Reconstruction

According to collection records of specimens and published reliable literature [25,27,31], we summarized host plants of other 18 species in *Mollitrichosiphum*. Except for the host plant of *M. kazirangi* still unknown, the hosts of all other species related to 14 families were listed in Table 1. Host plants of *Metatrichosiphon* have high diversity, referring to all 14 families mentioned above. While in the subgenus *Mollitrichosiphum*, three species are all restricted on Fagaceae and just a few samples of widespread species, *M. tenuicorpus* feed on plants of Betulaceae and Sabiaceae.

**Figure 3:**
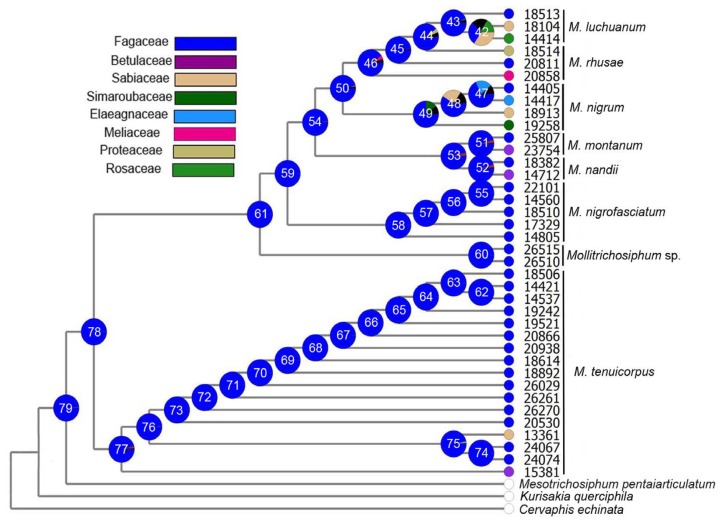
Ancestral state reconstruction of host plants in *Mollitrichosiphum*. The topology is derived from the ML tree of Figure 1A. Pie charts indicate the relative likelihoods at respective nodes. Terminal taxa are color-coded for the state of host use. The black part in the pie represents probability lower than 5%.

The results of ancestral state reconstruction suggest that Fagaceae was highly favored among all host plants. Node 61 and 77 (Figure 3), which are the MRCAs of the subgenus *Metatrichosiphon* and *Mollitrichosiphum*, showed high proportion of Fagaceae, 0.99 and 0.97, respectively; thereby the common ancestor of *Mollitrichosiphum* (node 78, 0.99) feeds on Fagaceae received strong support.

### 2.2. Discussion

#### 2.2.1. Phylogenetic Relationships

The monophyly of the genus *Mollitrichosiphum* was supported by phylogenetic analyses based on different approaches and genes (Figure 1). The phylogenetic trees of the combined dataset supported the division of the two subgenera (*Mollitrichosiphum* and *Metatrichosiphon*) based on morphological characters [24,25]. The molecular phylogenetic relationships between the species were also supported by morphological characters, such as the ultimate rostral segments of *M. luchuanum*, *M. rhusae* and *M. nigrum*, which are at least twice the length of the second hind tarsal segment, while the ultimate rostral segment of *M. nandii* and *M. montanum* are not more than twice the length of the second hind tarsal segment.

The phylogenetic placement of *M. nigrofasciatum* with respect to the other species remains undetermined, as the results based on different datasets were inconclusive. A possible explanation for the incongruent patterns of *M. nigrofasciatum* is incomplete lineage sorting during successive speciation events. However, more data and further study are needed to verify the real reasons that contribute to these results. Thus, additional markers are required for future studies to reveal the position of *M. nigrofasciatum*. Analysis of the EF-1α gene yielded a paraphyletic clade that included *M. luchuanum* and *M. rhusae*, while the combined datasets resulted in independent clades of the two species. This finding may be explained by the differential effectiveness of the genes. The nuclear EF-1α gene is usually used as a marker to investigate the phylogenetic relationship of aphids above the genus level, due to its slow rate of evolution, whereas the mitochondrial genes are more suitable for revealing the relationships at intra- and interspecies levels [32]. *M. luchuanum* and *M. rhusae* may have diverged recently, therefore, only mitochondrial genes may provide phylogenetic signals.

#### 2.2.2. Divergence Times and Biogeography

Southern China, which appears to be a center of diversity for *Mollitrichosiphum*, has experienced great topographical and climatic changes during the past million years. Estimated divergence times of this genus provide a reasonable temporal framework to reconstruct historical patterns of species divergence and distribution. Despite a degree of controversy regarding the exact timing of the uplift of the Tibetan Plateau [33], geological estimates date the period of rapid uplift unequivocally within the Late Tertiary/mid-Miocene [34], and episodes of uplift probably continued throughout the Late Pliocene (*c*. 3 mya) and into the Quaternary [5,35]. Clade A within *M. tenuicorpus* (Figure 2) is only currently distributed throughout the QTP, for which the divergence time was at approximately 11.0 mya, thereby falling within the time frame of the mid-Miocene uplift of the QTP. The clade including *M. nandii* and *M. montanum* was restricted to the Hengduan Mountains, which are located in the southeastern region of the QTP, and diverged from other species at approximately 10.6 mya (HPD: 6.2–15.1 mya). This result indicates that the distribution of these two species was probably also influenced by the uplift of the QTP.

The divergence of clades B and C within *M. tenuicorpus* was estimated at approximately 8.2 mya (Figure 2). These two clades are distributed east or west side of the Lancang River [30], which concurs with the long-held notion that the “Mekong-Salween Divide” (MSD) acted as a significant historical and geographical barrier to species dispersal [18,36,37]. The rivers located at the southeast margin of the Tibetan Plateau, the Dadu River, Mekong (Lancang River), Salween (Nu River), and Tsangpo-Brahmaputra (Yalu-Tsangpo River), were once tributaries of a single southward flowing river system, the Paleo-Honghe (Red River). The uplift of the Tibetan Plateau dramatically changed the characteristics of the major river drainages [38]. Time scales of formation of the MSD have rarely been estimated [18], thereby providing little insight into whether there is a direct link between the divergence of the B and C clades with the formation of the MSD. However, these historical events may have isolated the populations of *M. tenuicorpus* and stimulated differentiation among the populations.

*M. rhusae* and *Mollitrichosiphum* sp. are restrictedly distributed in the Hainan Island and Taiwan Island, respectively. The MRCAs of these two species are estimated at approximately 3.9 mya and 15.8 mya, respectively. Based on the results of geological studies, Hainan Island was first separated from the mainland of China during the Early Pleistocene (*c*. 2 mya) [7], and Taiwan Island was separated approximately 5 mya [39]. The divergence times show that the MRCAs of *M. rhusae* and *Mollitrichosiphum* sp. originated much earlier than the separation of the two islands. These results indicate that the geographical isolation of the two islands was probably not the primary factor triggering the differentiation of the two species. However, the isolation may have limited the subsequent species dispersal between the islands and mainland and thereby promoted the present distribution pattern. Although both islands were connected to the mainland when the sea level retreated during the Pleistocene, it has been suggested that the flora covering the emerged areas during the glaciations were poorly developed [40] and not suitable for aphid species dependent on woody host plants.

Fossils of *Mollitrichosiphum* have been found exclusively in Europe, while all extant species of this genus are currently restricted to Southeast Asia [25,41]. Peñalver *et al*. [42] demonstrated that the geographic ranges of Greenideinae were markedly wider during the Miocene and comprised the northern coasts of the Tethys Sea. During the Lower and Middle Miocene, the southern region of Europe was characterized by a hot subtropical climate. The terrestrial plant communities of Europe were dominated by needle-leaved forests, Cupressaceae species, and some broadleaf forest species [41]. Later geological events, such as the uplift of the Tibetan Plateau, affected both the climatic conditions of southern Europe and the composition of the flora [43]. During this period, a suitable climate and host plants for *Mollitrichosiphum* disappeared [44,45]. However, the climate in Southeast Asia remained unchanged. This may have determined the current distribution pattern of *Mollitrichosiphum* in southeastern Asia, including southern China.

#### 2.2.3. Species Differentiation of Chinese *Mollitrichosiphum*

Speciation processes are generally driven by the advent of barriers to gene flow between interbreeding populations [46,47]. The life history of many phytophagous insects often depends on specific host plants. Differences in host plant preference can result in reproductive isolation [48]. For example, studies of the pea aphid *Acyrthosiphon pisum* demonstrated that species differentiation occurred among different populations that fed on pea, clover and alfalfa [49,50].

Phylogenetic analyses show that *Mollitrichosiphum* sp. and *M. nigrofasciatum* are two basal clades in the subgenus *Metatrichosiphon*; together with *M. tenuicorpus*, these three species mainly feed on Fagaceae. The divergence times of these species were 15.8 mya, 10.6 mya and 18.4 mya, respectively (Figure 2), which are much earlier than the other species. In contrast, the host plants of a younger clade (with an age of 7.7 mya) including *M. nigrum*, *M. rhusae* and *M. luchuanum* are distinct (Table 1), and each of the three aphid species has hosts belonging to at least two plant families. Similarly, the sister species *M. nandii* and *M. montanum* with divergence times of 3.5 mya and 1.5 mya, are associated with host plants belonging to three and four families, respectively. It seems that these species have acquired a wider range of host plants during their evolutionary history.

The phylogenetic pattern of host association shows that basal species much more exclusively feed on Fagaceae, which is a common host plant for most species. Results of ancestral state reconstruction also supported the fact that Fagaceae may be the ancestral host plant of *Mollitrichosiphum*. Notably for the subgenus *Metatrichosiphon*, the acquisition of new hosts and the expansion of the host range might be the main factor driving species differentiation. Of the species in the subgenus *Mollitrichosiphum*, *M. tenuicorpus* is widely distributed in Southeast Asia and southern China, while the other three species are restrictedly distributed in Nepal, the northeastern region of India (Sikkim), and the southern edge of the QTP [25,27,31]. The divergence of the three clades within *M. tenuicorpus* (Figure 2) may be due to the combined effects of geographical isolation (as discussed above) and the differentiation of host plants (clade A on Betulaceae, clade B on Sabiaceae and Fagaceae, and clade C on Fagaceae). However, it seems that geographical isolation probably represents the key factor promoting the species divergence of the subgenus *Mollitrichosiphum*.

## 3. Experimental Section

### 3.1. Taxa Sampling

We sampled the Chinese *Mollitrichosiphum* species except for four species with very restricted distributions (*M. glaucae*, Hong Kong; *M. niitakaensis*, and *M. taiwanum*, Taiwan; *M. yamabiwae*, Taiwan, Hong Kong and Fujian). *Mollitrichosiphum* sp. is an undescribed species found in Taiwan Island (unpublished data), and can be distinguished from all other species of *Mollitrichosiphum* by an enlarged distal half of siphunculi. Specimens for slide-mounting were stored in 75% ethanol for morphological examination. Samples for molecular experiments were stored in 95% ethanol. All the samples were preserved at −30 °C. On the basis of current knowledge of the phylogenetic relationships within Aphididae [51,52], outgroups were chosen from *Cervaphis* van der Goot (Cervaphidini), *Eutrichosiphum* Essig & Kuwana (Greenideini), *Greenidea* Schouteden (Greenideini) and *Kurisakia* Takahashi (Thelaxinae), respectively. All voucher specimens were deposited in the National Zoological Museum of China (NZMC), Institute of Zoology, Chinese Academy of Sciences (Beijing). Collection information for all samples, including collection localities, host plants and collection dates are listed in Table 2.

### 3.2. DNA Extraction, PCR and Sequencing

CTAB DNA isolation technique [53] was used to extract genomic DNA. A 700 bp fragment of COI was amplified using primers LepF (5′-ATTCAACCAATCATAAAGATATTGG-3′) and LepR (5′-TAAACTTCTGGATGTCCAAAAAATCA-3′) from Foottit *et al*. [54]. About 800bp of Cytb was amplified using primers Cp1 (5′-GATGATGAAATTTTGGATC-3′) and Cp2 (5′-CTAATGCAATAACTCCTCC-3′) from Harry *et al*. [55]. The primers for amplifying EF-1α were EF3 (5′-GAACGTGAACGTGGTATCAC-3′) and EF2 (5′-ATGTGAGCAGTGTGGCAATCCAA-3′) [56]. PCRs (Polymerase Chain Reaction-amplification) for Cytb was performed in 30 μL reaction volumes: 3 μL 10 × PCR buffer, 2.4 μL dNTPs, 20 μL dd H_2_O, 0.4 unit Taq DNA polymerase (all from TransGen Biotech, Beijing, China) and 0.6 μL 10 μM forward and reverse primers (synthesized by Invitrogen Biotech, Shanghai, China). PCR volumes for COI were the same as that for Cytb, except for 19.4 μL dd H_2_O and 0.8 μM primers. The PCR amplification cycling conditions for COI were as follows: 95 °C for 5 min; 34 cycles including denaturation at 95 °C for 0.5 min, annealing at 52 °C for 0.5 min, and extension at 72 °C for 1 min; followed by 72 °C for 10 min. Thermocycling program for Cytb consisted of 95 °C for 3 min; 35 cycles of 92 °C for 1 min, 48 °C for 1.5 min and 72 °C for 1 min. A final extension step of 10 min at 72 °C was added after cycling. PCR thermal regime for EF-1α was initial denaturation at 95 °C for 4 min, 35 cycles including denaturation at 94 °C for 1 min, annealing at 50 °C for 1 min, and extension at 72 °C for 1.5 min, and final extension at 72 °C for 10 min. Sequencing reactions were performed using the same primers that were used for PCRs using ABI 3730 automated sequencer (Applied Biosystems, Foster City, CA, USA).

### 3.3. Sequence Alignment and Phylogenetic Analyses

The sequences were assembled using Seqman II (DNASTAR) and assessed manually. Multiple alignments were performed using ClustalX 1.81 [57]. Nucleotide composition, conserved sites, variable sites, and parsimony informative sites were calculated using MEGA 4 [58]. For the EF-1α gene, we used the coding regions in the phylogenetic analyses. The intronic sequences were removed using the GT-AG rule and by comparing the sequences with the cDNA sequence of *Mollitrichosiphum montanum* (GenBank accession number: DQ493826). The combined dataset of the COI, Cytb and EF-1α genes was evaluated with the partition homogeneity test implemented with PAUP* 4.10b [59] using random taxon addition (10 replicates), tree bisection-reconnection branch swapping, and heuristic searches with 100 random repartitions of the data.

Phylogenetic analyses were conducted in PAUP* 4.10b and MrBayes 3.1.2 [60]. Maximum parsimony (MP) analysis was conducted for all datasets independently, EF-1α and the combined datasets of COI + Cytb and COI + Cytb + EF-1α, using a heuristic search with 1000 random sequence repetitions and tree-bisection-reconnection (TBR) branch swapping. Consensus trees (50% majority rule) were obtained if more than one equally maximum parsimonious tree was found. The reliability of the MP trees was tested using the bootstrap approach [61] with 1000 pseudoreplicates using the heuristic search strategy and 100 random additions of sequences in each pseudoreplicate. Modeltest 3.7 [62] was used to determine the best-fit nucleotide substitution model for maximum likelihood (ML) analyses. The best models selected according to the Akaike Information Criterion (AIC) [63] for the combined dataset of mitochondrial genes and EF-1α, the combined mitochondrial dataset, and EF-1α were GTR + I + G, GTR + G and GTR + G, respectively. ML analysis was performed using PHYML [64], and nodal support among branches was evaluated by bootstrap analysis. Bayesian analysis was performed separately on the EF-1α gene and the combined dataset of three genes using the models selected by Modeltest 3.7. The combined dataset was partitioned into mitochondrial *versus* nuclear sequences and with separate models. Four chains were run starting from a random tree and proceeded for one million Markov chain Monte Carlo generations with tree sampling every 100 generations. Four independent runs were conducted to verify the results. The first 2500 trees (25% of the total) were discarded as burn-in. Next, 50% majority-rule consensus trees were generated from the remaining trees. The examination of the log-likelihood scores and the average standard deviation of the split frequencies suggested that the burn-in periods were long enough for chains to become stationary.

Results of the partition-homogeneity test indicated that nuclear *versus* mitochondrial alignments were incongruent (*P* = 0.01). However, because each gene provided similar tree topology, we still performed phylogenetic analyses based on the combined dataset. For Bayesian analysis, partitions were made for each gene separately using their own parameters derived from Modeltest.

### 3.4. Divergence Time Estimation and Ancestral State Reconstruction

To date, the only fossil species of this genus was *Mollitrichosiphum rubusensis* [41], which was found in Europe and dates back to 18–19 mya (million years ago). This time was used as a calibration point representing the minimum age of the most recent common ancestors (MRCAs) of this genus in our study. The divergence times among species were estimated using BEAST v1.6.1 [65] under a relaxed molecular clock and uncorrelated lognormal model. The GTR model of nucleotide substitution with gamma rate heterogeneity among sites and a proportion of invariant sites and Yule speciation process were used.

After determining the optimal parameters over several preliminary runs, multiple analyses were run for 10 million generations, and 25% of samples were removed as burn-in. The results were visualized in Tracer v1.5 [66], where the stationarity was assessed by ensuring that the Effective Sample Size (ESS) values were high for most parameters. The results proved repeatable over multiple independent runs, and thus a chronogram was reconstructed based on the results from a representative single run and was subsequently visualized using the program Fig Tree v1.3.1 [67].

Host plants of the genus *Mollitrichosiphum* were reconstructed according to a Bayesian criterion, using RASP 2.0 (Reconstruct Ancestal State in Phylogenies) software to implements Bayesian Binary MCMC (BBM). BBM offers a statistical procedure for inferring states, including geographic distributions, at ancestral nodes using a full hierarchical Bayesian approach [68]. For reconstruction of the ancestral state of host plants, each possible host plant of the species was coded based on the previous plants they fed on.

## 4. Conclusions

The monophyly of *Mollitrichosiphum* and most inter-species phylogenetic relationships were supported by molecular data as well as morphological characters. The sister relationships of *M. nandii* and *M. montanum*, *M. luchuanum*, *M. rhusae* with *M. nigrum* were supported in all analyses. Based on the divergence times, distribution and host plant information of the species, we speculate that geographical isolation due to the uplift of the Qinghai-Tibetan Plateau and the separation of the Hainan and Taiwan islands, as well as the expansion of host plant range may be the main factors driving differentiation of Chinese *Mollitrichosiphum* species and shaping their current distribution patterns.

## Supplementary Materials



## Figures and Tables

**Table 1:** Host plants of eighteen species in the genus *Mollitrichosiphum.*

Subgenera	Species		Host plants (Family)
*Mollitrichosiphum*	*M. godavariense*	1	Fagaceae

	*M. nigriabdominalis*	1	Fagaceae

	*M. tenuicorpus*	3	Fagaceae

			Betulaceae

			Sabiaceae

	*M. trilokum*	1	Fagaceae

*Metatrichosiphon*	*M. buddleiae*	2	Buddlejaceae

			Betulaceae

	*M. kazirangi*	1	unknown

	*M. montanum*	3	Betulaceae

			Juglandaceae

			Fagaceae
	
	*M. nandii*	4	Betulaceae

			Lauraceae

			Sapindaceae

			Fagaceae

	*M. rhusae*	4	Anacardiaceae

			Proteaceae

			Meliaceae

			Fagaceae

	*M. elongatum*	1	Fagaceae

	*M. syzygii*	1	Myrtaceae

	*M. luchuanum*	2	Rosaceae

			Fagaceae

	*M. nigrofasciatum*	1	Fagaceae

	*M. nigrum*	3	Sabiaceae

			Simaroubaceae

			Elaeagnaceae

	*M. yamabiwae*	1	Sabiaceae

	*M. niitakaensis*	1	Fagaceae

	*M. glaucae*	1	Fagaceae

	*M. taiwanum*	1	Sabiaceae

	*Mollitrichosiphum* sp.	1	Fagaceae

**Table 2:** *Mollitrichosiphum* samples examined in this study and related information.

Species	Voucher No.	Locality	Dates	Host plant	GenBank accession Nos: COI/Cytb/EF-1α
**Ingroups**					
*M. tenuicorpus* (Okajima)	20938	Hainan: Jianfengling Mt.	25 March 2008	*Castanopsis fabri* (Fagaceae)	JF969334/JF969383/JN645046
	13361	Yunnan: Baoshan City	20 September 2002	*Meliosma rigida* (Sabiaceae)	JF969308/JF969357/JN645020
	24074	Yunnan: Ruili City	2 December 2009	*Castanopsis calathiformis* (Fagaceae)	JF969339/JF969388/JQ418332
	24067	Yunnan: Ruili City	3 December 2009	*Castanopsis calathiformis* (Fagaceae)	JF969343/JF969392/JQ418331
	14421	Fujian: Wuyishan Mt.	6 July 2003	*Castanea* sp. (Fagaceae)	JF969311/JF969360/JN645042
	14537	Fujian: Wuyishan Mt.	19 July 2003	*Castanopsis sclerophylla* (Fagaceae)	JF969313/JF969362/JN645043
	18506	Hainan: Diaoluoshan Mt.	29 March 2006	*Cyclobalanopsis neglecta* (Fagaceae)	JF969321/JF969370/JQ418327
	19242	Hainan: Changjiang County	26 March 2007	Fagaceae	JF969327/JF969376/JN645045
	20866	Hainan: Jianfengling Mt.	15 December 2007	Fagaceae	JF969333/JF969382/JN645030
	22152	Fujian: Nanjing County	23 November 2008	unknown	JF969335/JF969384/JN645037
	22155	Fujian: Huboliao District	23 November 2008	unknown	JF969336/JF969385/JN645038
	19521	Hainan: Jianfengling Mt.	16 November 2006	*Quercus* sp. (Fagaceae)	JF969329/JF969378/JN645048
	22166	Fujian: Huboliao District	25 November 2008	unknown	JF969337/JF969386/JN645039
	26029	Guangxi: Huaping County	1 November 2010	*Castanopsis eyrei* (Fagaceae)	JN644999/JN645015/JN645054
	26261	Guangxi: Shiwandashan Mt.	19 November 2010	*Castanopsis fargesii* Franch. (Fagaceae)	JN645000/JN645016/JN645050
	22161	Fujian: Huboliao District	24 November 2008	unknown	JN644997/JN645013/JN645041
	24964	Guangxi: Damin Mt.	28 May 2011	Fagaceae	JQ418333
	18614	Guangdong: Chebaling District	13 April 2006	*Castanopsis carlesii* (Fagaceae)	JF969347/JF969396/JN645049
	20530	Yunnan: Simao City	27 July 2007	*Castanopsis ferox* (Fagaceae)	JF969330/JF969379/JN645028
	15381	Tibet: Motuo County	14 August 2003	*Alnus cremastogyne* (Betulaceae)	JF969317/JF969366/JN645044
	18892	Guangxi: Guilin City	18 May 2006	Fagaceae	JF969348/JF969397/JN645025
	26892	Fujian: Wuyishan Mt.	15 June 2011	Fagaceae	JQ418339
	26270	Guangxi: Shangsi Country	20 November 2010	Fagaceae	JQ418313/JQ418317/JQ418334
	26906	Fujian: Jiangle Country	17 June 2011	Fagaceae	JQ418340
*M. nigrum* (Zhang & Qiao)	14417	Fujian: Wuyishan Mt.	6 July 2003	*Elaeagnus pungens* (Elaeagnaceae)	JF969310/JF969359/JN645052
	19258	Guangxi: Xingan County	2 July 2006	*Ailanthus altissima* (Simaroubaceae)	JF969328/JF969377/JN645027
	21845	Hunan: Mangshan Mt.	17 July 2008	unknown	JF969341/JF969390/JN645032
	21856	Guangdong: Nanling Mt.	18 July 2008	unknown	JF969342/JF969391/JN645033
	18913	Guangxi: Longsheng County	21 May 2006	*Meliosma cuneifolia* (Sabiaceae)	JF969326/JF969375/JN645026
	21872	Guangdong: Nanling Mt.	19 July 2008	unknown	JN644995/JN645011/JN645035
	14405	Fujian: Wuyishan Mt.	4 July 2003	*Castanea* sp.(Fagaceae)	JN644988/JN645004/JN645019
*M. nandii* (Busu)	14712	Yunnan: Baoshan City	27 October 2003	*Alnus cremastogyne* (Betulaceae)	JF969315/JF969364/JQ418318
	18382	Tibet: Tongmai District	31 August 2005	*Fagus longipetiolata* (Fagaceae)	JF969320/JF969369/JQ418323
*M. nigrofasciatum* (Maki)	14560	Fujian: Wuyishan Mt.	20 July 2003	*Lithocarpus glaber* (Fagaceae)	JF969314/JF969363/JN645022
	14805	Fujian: Wuyishan Mt.	25 May 2004	*Cyclobalanopsis glauca* (Fagaceae)	JF969346/JF969395/JQ418319
	22101	Fujian: Liangyeshan Mt.	14 November 2008	*Lithocarpus glaber* (Fagaceae)	JF969351/JF969400/JN645036
	26742	Zhejiang: Anji Country	29 May 2011	Fagaceae	JQ418338
	21916	Guangdong: Nanling Mt.	21 July 2008	unknown	JN645047
	18510	Hainan: Lingshui Mt.	30 March 2006	*Lithocarpus elmerrillii* (Fagaceae)	JN644994/JN645010/JQ418328
	26275	Guangxi: Shiwandashan Mt.	20 November 2010	unknown	JN645001/JN645016/JN645051
	17329	Zhejiang: Taishun County	29 July 2005	*Quercus* sp. (Fagaceae)	JN644990/JN645006/JQ418322
	26733	Zhejiang: Anji Country	29 May 2011	Fagaceae	JQ418337
	18502	Hainan: Lingshui County	28 March 2006	*Castanopsis fabri* Hance (Fagaceae)	JN644993/JN645008/JQ418326
	17331	Zhejiang: Taishun County	29 July 2005	Fagaceae	JN645023
	21733	Hunan: Leiling County	7 July 2008	unknown	JN645031
	21859	Guangdong: Nanling Mt.	19 July 2008	unknown	JN645034
*M. rhusae* (Ghosh)	18513	Hainan: Diaoluoshan Mt.	30 March 2006	Fagaceae	JF969324/JF969373/JQ418329
	20811	Hainan: Wuzhishan Mt.	7 December 2006	Fagaceae	JF969331/JF969380/JQ418330
	20858	Hainan: Diaoluoshan Mt.	13 December 2007	Meliaceae	JF969332/JF969381/JN645029
*M. luchuanum* (Takahashi)	14414	Fujian: Wuyishan Mt.	6 July 2003	*Amygdalus persica* (Rosaceae)	JF969309/JF969358/JN645021
	18104	Fujian: Wuyishan Mt.	24 October 2005	*Meliosma rigida* (Sabiaceae)	JF969319/JF969368/JN645024
*M. montanum* (van der Goot)	16504	Tibet: Zhangmu County	27 July 2005	unknown	JF969318/JF969367/JQ418321
	23754	Yunnan: Qingliang County	10 November 2009	*Alnus nepalensis* (Betulaceae)	JF969338/JF969387/JN645040
	18324	Tibet: Linzhi City	22 August 2005	unknown	JF969344/JF969393/JN645018
	25807	Tibet: Zhangmu County	8 August 2010	Fagaceae	JN644998/JN645012/JN645053
*Mollitrichosiphum* sp.	26510	Taiwan: Taman Mt.	14 June 2011	Fagaceae	JN645002/JQ418315/JQ418335
	26515	Taiwan: Hualian County	20 June 2011	Fagaceae	JN645003/JQ418316/JQ418336
**Outgroups**					
*Eutrichosiphum tattakanum* (Takahashi)	17184	Sichuan: Miyi County	20 April 2005	unknown	JN645055
*Kurisakia querciphila* (Takahashi)	16201	Guizhou: Leigongshan Mt.	31 May 2005	Fagaceae	JF969355/JF969404/JQ418320
*Mesotrichosiphum pentaiarticulatum* (Zhang & Qiao)	18489	Hainan: Lingshui Mt.	27 March 2006	Fagaceae	JQ418312/JQ418314/JQ418325
*Cervaphis echinata* (Hille Ris Lambers)	18461	Hainan: Jianfengling Mt.	21 March 2006	*Paulownia* sp. (Scrophulariaceae)	JF969356/JF969405/JQ418324

**Table 3:** Characters of DNA sequences and the datasets used in phylogenetic analyses.

	Individual datasets			Combined dataset
		
	COI a	Cytb b	EF-1α c	
Number of taxa	72	57	59	50
Aligned sequence length (bp)	606	665	785	2056
Conserved sites	435 (473)	450 (498)	596 (656)	1500 (1644)
Variable sites	171 (133)	215 (167)	189 (129)	556 (412)
Parsimony-informative sites	147 (128)	141 (134)	111 (73)	365 (328)

Numbers in the bracket are the results of ingroups;

a COI: Cytochrome C Oxidase Subunit I;

b Cytb: Cytochrome b;

c EF-1α: Elongation Factor-1α.
