# Supplementary material for: Species Differentiation of Chinese Mollitrichosiphum (Aphididae: Greenideinae) Driven by Geographical Isolation and Host Plant Acquirement
Source: Int J Mol Sci. 2012 Aug 21;13(8):10441–60. doi: 10.3390/ijms130810441 (PMC3431871; doi:10.3390/ijms130810441)
Supplement: Supplementary file 1 [file ijms-13-10441-s001.pdf]

Supplementary Material

Table S1. Distribution of species in present study of the genus *Mollitrichosiphum*.

Subgenus	Species	Distribution
Mollitrichosiphum	*M. godavariense*	**Nepal**: Kathmandu (Godawari)
	M. nigriabdominalis	**India**: Sikkim (Sanklang)
	M. tenuicorpus	**China**: Hainan (Jianfengling, Diaoluoshan, Changjiang), Yunnan (Baoshan, Ruili, Simao), Fujian (Wuyishan, Nanjing, Huboliao, Jiangle), Guangxi (Huaping, Shiwandashan, Damingshan, Guilin, Shangsi), Guangdong (Chebaling), Tibet (Motuo), Taiwan (Suisha, Botan);<br>**India**: Meghalaya (Shillong), Sikkim (Gangtok, Sanklang);<br>**Indonesia**: Java;<br>**Japan**; **Korea**; **Thailand**
	M. trilokum	**India**: Sikkim (Gangtok)
Metatrichosiphon	*M. buddleiae*	**India**: Uttar Pradesh (Gourikund), Sikkim (Pelling);<br>**Nepal**: Nagarkot
	M. kazirangi	**India**: Assam (Kaziranga sanctuary)
	M. montanum	**China**: Tibet (Zhangmu, Linzhi), Yunnan (Qingliang);<br>**India**: Meghalaya, Uttar Pradesh (Kausani), West Bengal (Durbin, Kalimpong), Sikkim (Gangtok);<br>**Nepal**: Godawari, Nagarkot, Nagarjun, Narayansthan
	M. nandii	**China**: Yunnan (Baoshan), Tibet (Tongmai, Motuo), Sichuan (Xichang);<br>**India**: West Bengal (Kalimpong), Sikkim
	M. rhusae	**China**: Hainan (Lingshui, Wuzhishan, Diaoluoshan,);<br>**India**: Meghalaya (Shillong)
	M. elongatum	**Indonesia**: Java
	M. syzygii	**Indonesia**: Java

Table S1. *Cont.*

Subgenus	Species	Distribution
Metatrichosiphon	*M. luchuanum*	**China**: Fujian (Wuyishan), Guangdong (Nanling)
	M. nigrofasciatum	**China**: Fujian (Wuyishan, Liangyeshan), Guangdong (Chebaling, Nanling), Hainan (Lingshui), Hunan (Leiling), Guangxi (Shiwandashan), Zhejiang (Anji, Taishun); **Japan**
	M. nigrum	**China**: Fujian (Wuyishan), Guangxi (Xingan, Longsheng), Hunan (Mangshan), Guangdong (Nanling)
	M. yamabiwae	**China**: Fujian (Wuyishan), Taiwan, Hong Kong
	M. niitakaensis	**China**: Taiwan
	M. glaucae	**China**: Hong Kong
	M. taiwanum	**China**: Taiwan; **Japan**
	Mollitrichosiphum. sp	**China**: Taiwan (Tamanshan, Hualian)

© 2012 by the authors; licensee MDPI, Basel, Switzerland. This article is an open access article distributed under the terms and conditions of the Creative Commons Attribution license (http://creativecommons.org/licenses/by/3.0/).
